# Protocol for a systematic review of N-of-1 trial protocol guidelines and protocol reporting guidelines

**DOI:** 10.1186/s13643-017-0525-4

**Published:** 2017-07-06

**Authors:** Antony J. Porcino, Salima Punja, An-Wen Chan, Richard Kravitz, Aaron Orkin, Philippe Ravaud, Christopher H. Schmid, Sunita Vohra

**Affiliations:** 1 CARE Program, Department of Pediatrics, Faculty of Medicine and Dentistry, and Integrative Health Institute, University of Alberta, Suite #1702, College Plaza 8215 112 St. NW, Edmonton, AB T6G 2C8 Canada; 2 Women’s College Research Institute, Women’s College Hospital, Department of Medicine, University of Toronto, 76 Grenville St, Toronto, ON M5S 1B1 Canada; 3 UC Davis Department of Internal Medicine, 4150 V St., Sacramento, CA 95817 USA; 4 Dalla Lana School of Public Health, University of Toronto, 155 College Street, Toronto, ON M5T 1P8 Canada; 5 EQUATOR France, Centre d’épidémiologie clinique, Hôpital Hôtel-Dieu, 1 place du Parvis Notre-Dame, Paris, 75004 France; 6 Center for Evidence Based Medicine, School of Public Health, Brown University, 121 South Main Street, 8th Floor, Providence, RI 02903 USA

**Keywords:** Systematic review, N-of-1 trials, Clinical protocols, Guidelines, Single case experimental designs

## Abstract

**Background:**

N-of-1 trials are multiple cross-over trials done in individual participants, generating individual treatment effect information. While reporting guidelines for the CONSORT Extension for N-of-1 trials (CENT) and the Standard Protocol Items: Recommendations for Interventional Trials (SPIRIT) already exist, there is no standardized recommendation for the reporting of N-of-1 trial protocols.

**Objective:**

The objective of this study is to evaluate current literature on N-of-1 design and reporting to identify key elements of rigorous N-of-1 protocol design.

**Methods:**

We will conduct a systematic search for all N-of-1 trial guidelines and protocol-reporting guidelines published in peer-reviewed literature. We will search Medline, Embase, PsycINFO, CINAHL, the Cochrane Methodology Register, CENTRAL, and the NHS Economic Evaluation Database. Eligible articles will contain explicit guidance on N-of-1 protocol construction or reporting. Two reviewers will independently screen all titles and abstracts and then undertake full-text reviews of potential articles to determine eligibility. One reviewer will perform data extraction of selected articles, checked by the second reviewer. Data analysis will ascertain common features of N-of-1 trial protocols and compare them to the SPIRIT and CENT items.

**Discussion:**

This systematic review assesses recommendations on the design and reporting of N-of-1 trial protocols. These findings will inform an international Delphi development process for an N-of-1 trial protocol reporting guideline. The development of this guideline is critical for improving the quality of N-of-1 protocols, leading to improvements in the quality of published N-of-1 trial research.

**Electronic supplementary material:**

The online version of this article (doi:10.1186/s13643-017-0525-4) contains supplementary material, which is available to authorized users.

## Background

N-of-1 trials are a multiple cross-over trial design within a single participant, wherein a participant receives all treatments an equal number of times with at least two tests of each treatment (e.g., ABAB) [1–4]. They have been recommended for personalizing treatment since the late 1960s [5, 6]. Though useful for situations where substantial heterogeneity of treatment effects may be an issue, N-of-1 trials are especially important when large parallel group randomized controlled trials (RCTs) are not feasible and evidence is limited, such as for evaluation of rare diseases, many pediatric conditions, and patients with co-morbid conditions or multiple concurrent treatments. When done in series, these trials can also facilitate the development of rigorous evidence in those populations [7–11].

Due to the design’s multiple cross-overs within a single participant, some aspects of an N-of-1 trial require particular consideration for feasibility, including: (i) the types of conditions (e.g., best if chronic and stable; if episodic, then frequently occurring) or treatments (e.g., reversible, preferably with quick onset and quick offset) that may be evaluated through the N-of-1 design; (ii) some design constituents (e.g., shorter treatment period lengths preferable, presence or absence of washout); and (iii) opportunities for participant contribution during the design process (e.g., treatment and outcome choice preferences). Methods of analysis of a series of N-of-1 trials, including meta-analysis of N-of-1 data, continue to be refined, increasing their research value [12, 13].

Careful development of a clinical trial protocol is important for researchers, ethics review boards, funders, and journals [14, 15]. Protocol reporting guidelines ensure that the specifics of the planned research trial are reported in a transparent, accurate, standardized manner [14, 16]. For reporting trial protocols, the Standard Protocol Items: Recommendations for Interventional Trials (SPIRIT) guideline [15] provides recommendations for essential elements to address in a clinical trial protocol. A SPIRIT Extension for N-of-1 Trials (SPENT) will help trialists report the important details unique to this trial design. This synthesis of published guidelines on N-of-1 trial protocol design will be the first step in the development process of SPENT [16].

This systematic review will search for both N-of-1 protocol reporting guidelines as well as clinical trial design recommendations. A preliminary exploratory search suggested no N-of-1 protocol reporting guidelines. A number of articles detail methodological recommendations for N-of-1 trials [1, 17–23], including the Consolidated Standards of Reporting Trials (CONSORT) Extension for N-of-1 Trials (CENT) [8]. Furthermore, this review includes psychological as well as medical literature databases to increase identification of relevant N-of-1 design guidelines.

### Objectives

The purpose of this review is to systematically identify published guidelines and reporting guidelines relevant to N-of-1 trial protocols. The main goal is to identify a list of relevant N-of-1 trial protocol items that might be included in an N-of-1 protocol reporting guideline, and summarize similarities and gaps to items in the SPIRIT and CENT reporting guidelines.

## Methods and design

This systematic review will be done using recommendations from *Systematic Reviews* by the Centre for Reviews and Dissemination [24] and is based on the SPIRIT and CENT systematic review protocols [8, 14]. It is being reported using the 2015 Preferred Reporting Items for Systematic Reviews and Meta-Analyses: Protocols (PRISMA-P) statement items [25]. This protocol is not registered with the International Prospective Register of systematic reviews (PROSPERO) because a review of protocol guidelines does not meet the inclusion criteria [26].

### Criteria for document inclusion

#### Types of documents

Published, peer-reviewed articles written in English will be included if they describe a guideline for developing and conducting an N-of-1 trial or for reporting an N-of-1 trial protocol.

#### Eligibility criteria for guidelines of interest

Potential articles will be eligible for inclusion if they contain an explicit, itemized guide detailing the content or headings for designing or reporting a complete N-of-1 trial protocol [14] or aspects of a such a protocol. Due to lack of specificity, articles that simply describe a generic ABAB trial design for the purpose of reviewing or contrasting different trial designs, or that pertain to other single subject research designs, will be excluded.

#### Information sources and their search strategies

Three strategies will be used for the search: (1) a systematic review of the literature; (2) searching references cited in the N-of-1 articles found by the search strategy; and (3) requests for guidelines specific to N-of-1 trials will be sent to 31 large private and public research and funding organizations in North America, Europe, and Australia.

##### Systematic review of the literature

The following databases will be searched: Medline (Ovid interface, 1946 to Feb 2015), Embase (Ovid interface, 1974 to Feb 2015), PsycINFO (Ovid interface, 1806 to Feb 2015), CINAHL (EBSCOHost interface, 1982 to present), Cochrane Methodology register (Wiley interface, through to 2015), CENTRAL (Wiley interface, through to 2015), and the NHS Economic Evaluation Database (Wiley interface, coverage dates unstated). The search strategy for Medline can be found in Appendix 1. It will be modified as appropriate for the individual database search parameters.

This search protocol was developed in collaboration with a health research librarian, based on a previous N-of-1 trial systematic review protocol (used for the CENT guidelines [8]), the protocol used for the SPIRIT Guidelines [14], and a review of the keywords from several N-of-1 guidance documents [1, 20, 27]. The search protocol was validated for effectiveness [28] using a small set of well-established N-of-1 methodology documents [1, 18, 19, 22, 27, 29].

#### Data management

##### Screening

All search result references will be directly imported into DistillerSR®, an on-line program for systematic review data management and analysis. For Stage 1 screening, two reviewers (AP and SP) will independently scan titles and abstracts of all references to identify potentially relevant articles according to the inclusion/exclusion criteria. Articles meeting the inclusion criteria, and those where there is any uncertainty, will move to Stage 2 where full articles will be screened. The reviewers developed the screening questions for Stage 1 and 2 based on the inclusion/exclusion criteria, and tested the forms, resolving any discrepancies. For both stages, any differences of screening status between the two reviewers will be discussed, and a third referee (AO) will resolve remaining disagreements.

##### Data extraction

All articles selected from Stage 2 will be reviewed using a data extraction form to identify potential standard recommended N-of-1 protocol items. The form, reviewed by the team, includes all SPIRIT guideline items, CENT guideline items that are not addressed by SPIRIT, and additional items from our reference articles [1, 18, 19, 22, 29] that address key N-of-1 trial design issues not otherwise addressed in SPIRIT and CENT. Additionally, to learn more about the development of the included guidelines, we have included items from SPIRIT’s initial systematic review protocol that are specific to the development process of the guidelines. The screening form includes text boxes for additional description for N-of-1-specific items and a final open-ended text box for any additional notes on topics of relevance not in the screening list. Piloting of the data extraction form using the N-of-1 guidance documents will occur before undertaking the data extraction. The screening form is in Additional file 1; the key identifying each item’s reporting guideline source is available on request.

Because data extraction is focused on verifying if each item is present in the article, one reviewer will extract (AP), and a second (SP) will check a 15% sample from each paper for extraction accuracy. Any differences of data extraction between the two reviewers will be discussed, and a third referee (AO) will resolve remaining disagreements; if greater than 20% disagreement, full review will be considered.

#### Data analysis and synthesis

Extracted data will be used for descriptive statistical analysis (*n*, %, per item and per reporting topic headings in the data extraction form), and the median number of items per guideline. For items represented in at least 50% of the articles, frequencies of representation for each item in the SPIRIT and CENT statement items will be calculated to identify similarities, gaps, and topics not identified in SPIRIT and CENT. To identify other issues or items not addressed by the data extraction form, synthesis of any notes or comments made in the screening form will be assessed using basic qualitative descriptive analysis, in which core topics or ideas are tabulated and clustered, staying within the language and meaning of the note text [30, 31]. This analysis process will be directed by one reviewer (AP) and reviewed by a second. All protocol-specific results and extracted qualitatively-identified topics will form the starting list for the development of SPENT. This list will be assessed and refined using a sample of published and unpublished N-of-1 trial protocols, followed by an international Delphi process.

## Discussion

This systematic review will find and synthesize the recommendations on the development and publishing of N-of-1 trial protocols. The results will be reported using the Preferred Reporting Items for Systematic reviews and Meta-Analyses (PRISMA) Statement [32].

Within single subject research, N-of-1 is one of many different established trial designs. The associated terminology regarding these designs is becoming standardized [8, 23]. However, in older articles, “N-of-1” may have referred to any form of research in a single person. Conversely, an actual N-of-1 trial may not have used the explicit N-of-1 designation. Therefore other older and more generic terms are included in the search strategy.

The search strategies are not optimized to capture non-English N-of-1 terminology. However, many journals indexed in PubMed provide English abstracts and MeSH or key terms. A search for “n-of-1” and “n of 1” in the non-English languages included in PubMed found only eight articles, all N-of-1 trials, from 1989 to 2014. Several of these non-English language articles only cited English N-of-1 design articles referenced in this protocol; non-English language N-of-1 guidelines were not identified. It is therefore assumed that excluding non-English language articles will not likely exclude any key relevant knowledge from this systematic review.

Some medical epistemologists argue that N-of-1 RCTs offer the most rigorous form of evidence-based medicine by combining individual patient values, randomization, and clinician expertise into a single experiment or clinical decision-making event [2, 3, 33]. They may be a useful option for addressing the personalized care recommendations of the US Patient-Centered Research Institute (PCORI) and the Canadian Strategy for Patient-Oriented Research (SPOR) [3]. Despite these advantages and calls for the routine use of N-of-1 trials in clinical practice and decision-making, N-of-1 trials remain an underused method and strategy in research and practice. The broad scope of this systematic review will help provide an appropriate basis for developing rigorous N-of-1 trial protocols, and an N-of-1 trial protocol reporting guideline (SPENT). Specifically, the results will form the basis for the Delphi process needed to assess and develop possible items in SPENT. In turn, these should strongly support improvements in the implementation of patient-centred research and the quality of published N-of-1 trial research.

## Appendix 1 – Electronic search strategies

[mp = title, abstract, original title, name of substance word, subject heading word, keyword heading word, protocol supplementary concept word, rare disease supplementary concept word, unique identifier]

### MEDLINE

Medline 10 Feb 2015 skj

1. n-of-1.mp.
2. n of 1.tw.
3. (individual$ adj3 trial$).mp.
4. (individual$ adj3 test$).tw.
5. ((single or individual) adj (subject or patient or case) adj3 (trial$ or design)).tw.
6. individuali#ed medication effectiveness test$.tw.
7. patient$ as their own control$.tw.
8. abab.ti,ab.
9. or/1-8
10. Double-Blind Method/
11. Research Design/
12. Randomized Controlled Trials as Topic/
13. cross-over studies/
14. Placebos/
15. Guidelines as Topic/
16. bayes theorem/
17. frequentist.mp.
18. or/10-17
19. 9 and 18
20. limit 19 to humans
21. limit 20 to English language

## Additional file

Additional file 1: Level 3 Data Extraction Questions. (DOC 31 kb)

## Abbreviations

CENT: CONSORT Extension for N-of-1 trials
RCT: Randomized controlled trial
SPENT: SPIRIT Extension for N-of-1 trials
SPIRIT: Standard Protocol Items: Recommendations for Interventional Trials
