# Supplementary material for: Protocol for a systematic review of N-of-1 trial protocol guidelines and protocol reporting guidelines
Source: Syst Rev. 2017 Jul 6;6:132. doi: 10.1186/s13643-017-0525-4 (PMC5498872; doi:10.1186/s13643-017-0525-4)
Supplement: Additional file 1: — Level 3 Data Extraction Questions. (DOC 31 kb) [file 13643_2017_525_MOESM1_ESM.doc]

## Additional file 1: Level 3 Data Extraction Questions

Note: **“MRoD”** indicates the article **m**akes **r**ecommendations **o**r **d**iscusses the topic.

**TITLE and METADATA of the project**

MRoD the title (e.g., identify the study design (N-of-1),
population, intervention, trial acronym) no/yes

MRoD trial identification and registration number. no/yes

MRoD inclusion of the World Health Organization Trial Registration
Data Set no/yes

MRoD protocol version numbering? no/yes

MRoD specifying sources of funding, material, and other support no/yes

MRoD Roles and responsibilities of people involved with the trial no/yes

Number of contributors [text box]

Expertise of contributors [text box]

**INTRODUCTION section of the project**

MRoD developing the background and rationale of the project/
what should be included no/yes

MRoD the comparators used in a project no/yes

MRoD developing objectives or hypotheses of N-of-1 projects no/yes

Guideline development described no/yes

—If described, development process included (checkbox) — consensus

— expert opinion

— research evidence

— piloting

— validity testing — other [text box]

**TRIAL DESIGN recommendations**

MRoD regarding the design of single or multiple trials
[overview of process; specific items below] no/yes

MRoD on relationship between multiple trials
(staggered, baseline, multi-practitioner/site) no/yes

MRoD regarding trial design framework
(superiority, equivalence, non-inferiority, exploratory) no/yes

MRoD participation of the patient in design/implementation no/yes [+ text box]

**METHODS of a study**

MRoD the study setting no/yes

MRoD eligibility criteria? (checkbox) — not specified

— diagnosis/disorder

— diagnostic criteria

— co-morbid

condition(s)

— concurrent

treatment

— trial facilitators &

barriers

MRoD intervention dosing no/yes [+ text box]

MRoD intervention administration no/yes

MRoD washouts or lead-in/ramping up no/yes [+ text box]

MRoD co-treatments no/yes

MRoD planned number of phase pairs/trios no/yes [+ text box]

MRoD criteria for discontinuing/modifying tx (individual) no/yes

MRoD issues of individualizing tx for an individual in a series design no/yes

MRoD adherence no/yes

MRoD continuation and care for other medical conditions during the trial no/yes

MRoD choice of outcome measures no/yes [+ text box]

MRoD preparing a timeline no/yes

MRoD regarding sample size no/yes

MRoD recruitment process no/yes

**ASSIGNMENT OF INTERVENTIONS**

MRoD randomizing process or treatment sequence no/yes [+ text box]

MRoD concealing/blinding the allocation sequence no/yes [+ text box]

MRoD implementation of the allocation sequence (who, how) — none

(select one only, radio button) — who

— how

— both

MRoD allocation concealment/blinding or non-blinding (checkbox) — none

— blinding needed

— blinding protocol

— no-blinding option

MRoD when blinded, when to unblind no/yes

**DATA COLLECTION, MANAGEMENT, and ANALYSIS**

MRoD procurement/collection of the outcome measures/data no/yes

MRoD increasing/promoting participant retention and what to do
with drop-out issues (tx, data loss) no/yes

MRoD data management no/yes

MRoD statistical analysis (checkbox) — none

— parametric

(t, chi-sq, etc.)

— regression

— Bayesian

— versus visual

— series synthesis

— issues: carry-over,

period, and intra-

subject correlation

— other [text box]

MRoD visual analysis (checkbox) — no

— yes

— celeration line

MRoD adjusted or subgroup analyses no/yes

MRoD non-adherence and missing data processes no/yes

**MONITORING**

MRoD data monitoring committee/process no/yes

MRoD interim analyses and stopping guidelines no/yes

MRoD monitoring or reporting of harms no/yes

MRoD auditing the trial no/yes

**ETHICS AND DISSEMINATION**

MRoD seeking ethics approval (checkbox) — no

— yes

— issue: clinical trials

MRoD management of protocol changes no/yes

MRoD obtaining consent no/yes

MRoD managing confidentiality throughout a trial no/yes

MRoD declaration of competing interests no/yes

MRoD management of access to data no/yes

MRoD ancilliary and post-trial care no/yes

MRoD dissemination of results no/yes

**APPENDICES**

MRoD informed consent materials given to participants/guardians no/yes

MRoD plans, if any, for collection, evaluation, and storage of
biological specimens no/yes

**OTHER ITEMS OF INTEREST/NOTE** [text box]
